# Visibility-Based Calibration of Low-Cost Particulate Matter Sensors: Laboratory Evaluation and Theoretical Analysis

**DOI:** 10.3390/s25226995

**Published:** 2025-11-16

**Authors:** Ayala Ronen

**Affiliations:** 1 Environmental Physics Department, Israel Institute for Biological Research (IIBR), Ness Ziona 7410001, Israel; ayalar@iibr.gov.il or ronenayala@gmail.com; 2 Department of Physics, Technion-Israel Institute of Technology, Haifa 3200003, Israel

**Keywords:** low-cost particulate matter sensors, air quality monitoring, visibility sensor, atmospheric extinction coefficient, gravimetric calibration

## Abstract

Low-cost optical sensors for particulate matter (PM) monitoring, such as the SDS011, are widely used due to their affordability and ease of deployment. However, their accuracy strongly depends on aerosol properties and environmental conditions, necessitating reliable calibration. This study presents a theoretical and laboratory evaluation of a practical calibration method based on visibility sensors, which measure atmospheric light extinction and are readily available at many meteorological stations. Experiments were conducted in a controlled aerosol chamber, using SDS011 sensors, visibility sensors (FD70 and SWS250), and gravimetric samplers. The mass extinction coefficient was determined through parallel measurements of visibility and mass concentration, enabling conversion of optical signals into accurate PM values. The calibrated SDS011 sensors demonstrated consistent response with a stable normalization factor (dependent on aerosol type, wavelength, and particle size), allowing their deployment as a spatially distributed sensor network. Comparison with manufacturer calibration revealed substantial deviations due to differences in aerosol optical properties, highlighting the importance of application-specific calibration. The visibility-based approach enables real-time, continuous calibration of low-cost sensors with minimal equipment, offering a scalable solution for PM monitoring in resource-limited or remote environments. The method’s robustness under varying environmental conditions remains to be explored. Nevertheless, the results establish visibility-based calibration as a reliable and accessible framework for enhancing the accuracy of low-cost PM sensing technologies. The method enables scalable calibration with a single gravimetric reference and is suited for future field deployment in resource-limited settings, following additional validation under real atmospheric conditions.

## 1. Introduction

Experiments involving aerosols, whether conducted in controlled chambers or in field studies, require integrating various real-time measurement methods to characterize aerosol properties. Among the key parameters commonly measured is the aerosol mass concentration.

A wide range of instruments for aerosol concentration measurements is commercially available and employs diverse technologies. However, scientific-grade equipment is often prohibitively expensive, limiting the feasibility of widespread environmental monitoring and creating a gap in the availability of affordable, simple, and accurate real-time measurement tools for various types of aerosols.

In recent years, there has been a significant rise in the use of low-cost particulate matter sensors (LCPMSs) for monitoring airborne particulate matter (PM) concentrations. Their operation is based on measuring the intensity of scattered light as particles pass through a sensing volume. An airstream carries aerosol particles into a light beam, where the particles scatter the incident radiation. A portion of the scattered light is detected by a photodiode placed at a fixed angle, typically 90° or 120°, relative to the beam axis. It is generally assumed that only a single particle is detected at a time, which sets an upper limit on the measurable particle concentration.

Since the signal generated is proportional to the detected light intensity, and thus to the particle size, analyzing the number and amplitude of detection events allows estimation of particle number and particle mass concentrations. These analog signals are then amplified, digitized, and converted into size or mass bins through a pre-calibrated response curve [1].

Due to their low cost, LCPMs can be deployed at high density, thereby facilitating real-time monitoring with broad spatial coverage [2]. However, their accuracy is strongly influenced by several factors, including particle characteristics (size, chemical composition, density, and refractive index) and environmental conditions (temperature, humidity, etc.) [3]. As a result, reliable operation of these sensors requires calibration procedures tailored to the specific experimental or environmental context.

Various studies have investigated methods for calibrating LCPMS. A commonly used approach involves collocating the sensor with a professional-grade reference monitor for a defined period and comparing outputs [4]. Light-scattering instruments such as nephelometers have also been explored for this purpose [5]. These instruments offer high precision and have a well-defined theoretical basis for converting scattered light into mass concentration.

However, nephelometers are expensive, delicate, and generally restricted to laboratory or regulatory use. They typically operate at a fixed or limited set of scattering angles and are sensitive to humidity, making them less suitable for low-cost, low-maintenance field deployments.

Other calibration strategies rely on controlled laboratory environments [6] or statistical, physical, or meteorological models. However, these approaches often involve assumptions about the measurement medium and may be prone to systematic errors.

Thus, the challenge of calibrating low-cost sensors calls for an efficient, independent, cost-effective method that does not require access to professional reference stations or high-end instruments.

In this study, we propose an alternative optical calibration method using a visibility sensor. Recent studies have shown the potential of visibility sensors as reliable optical instruments for estimating and correlating particulate matter concentrations under real atmospheric conditions [7,8]. Building on these findings, we propose an alternative visibility-based calibration approach for low-cost particulate matter sensors.

A visibility sensor measures forward-scattered light at a defined angular range and operates at a fixed wavelength. From the detected signal, the extinction coefficient is derived and converted to visibility, typically spanning a range from a few meters to several tens of kilometers. The method proposed here is based on calibrating the visibility sensor against aerosol mass concentration and subsequently using it to calibrate LCPMSs. The validity of this approach is supported, as both measurement methodologies fundamentally rely on the scattering of electromagnetic radiation.

Using a visibility sensor for calibrating LCPMSs enables a practical and scalable field calibration approach. These sensors are relatively low-cost and widely deployed in the transportation and aviation sectors. They operate continuously and offer robust performance. Therefore, this approach provides an accessible path for calibrating sensor networks in community monitoring projects or in remote areas lacking access to professional reference infrastructure. It contributes to the broader transition from theoretical research to scalable, sensor-based air quality monitoring.

This study aims to demonstrate the feasibility of establishing a reliable relation between optical extinction and gravimetrically measured particulate concentration under controlled laboratory conditions. While the approach is grounded in quantitative optical principles, it should at this stage be regarded as a semi-quantitative framework for sensor calibration, pending further validation in real field environments.

## 2. Materials and Methods

### 2.1. Theoretical Background: Optical Monitoring of Aerosol Concentration

The physical basis for optical aerosol sensing relies on the attenuation of electromagnetic radiation as it propagates through a medium containing suspended particles. This attenuation, described by the mass extinction coefficient (MEC), *α_ext_*, depends on the particle size distribution, the complex refractive index, and the wavelength of the probing light.

The Beer-Lambert Law [9] describes the exponential decay in light intensity as a function of path length through the medium. It relates the incident light intensity (*I*_0_) to the transmitted intensity (*I*) after passing through a path of length *L*, containing a particle concentration *c*, as follows:

(1) $$I=I_{0}e^{-\alpha_{ext}cL}.$$

When the particle radius *r* is on the same order of magnitude as the incident-light wavelength *λ*, both scattering and absorption contribute significantly to the overall extinction. This regime is accurately described by Mie theory [10], which provides analytical solutions for the scattering of electromagnetic waves by spherical particles.

Mie theory defines two components of the scattered electric field amplitude: parallel polarization (*S*_1_(*θ*)) and perpendicular polarization (*S*_2_(*θ*)), relative to the plane of polarization of the incident wave. These amplitudes are given by the following:

(2) $$S_{1}\left(\theta \right)=\sum_{n=1}^{\infty }\frac{2n+1}{n\left(n+1\right)}\left[a_{n}\pi_{n}\left(cos\theta \right)+b_{n}\tau_{n}(cos\theta )\right]\;S_{2}\left(\theta \right)=\sum_{n=1}^{\infty }\frac{2n+1}{n\left(n+1\right)}\left[a_{n}\tau_{n}\left(cos\theta \right){+b}_{n}\pi_{n}\left(cos\theta \right)\right]$$

where *a_n_* and *b_n_* are the Mie scattering coefficients, which depend on the size parameter and the complex refractive index *m* of the aerosol material. *π_n_* and *τ_n_* are angle-dependent functions derived from associated Legendre polynomials and describe the angular scattering pattern. The angular distribution of scattered intensity is described by the scattering phase function, which captures how scattering strength varies with scattering angle. The total scattered intensity, considering both polarizations, is given by the following:

(3) $$I\left(\theta \right)={S_{1}\left(\theta \right)}^{2}+{S_{2}\left(\theta \right)}^{2}.$$

MEC represents the aerosol attenuation per unit mass and is calculated using the following [11]:

(4) $$\alpha_{ext}=\frac{\int \pi r^{2}Q_{ext}n(r)dr}{\rho \int \frac{4}{3}\pi r^{3}n(r)dr},$$

where $Q_{ext}$ is the extinction efficiency factor, here computed based on [12], *n*(*r*) is the particle size distribution function, and ρ is the particle density.

**Figure 1:**
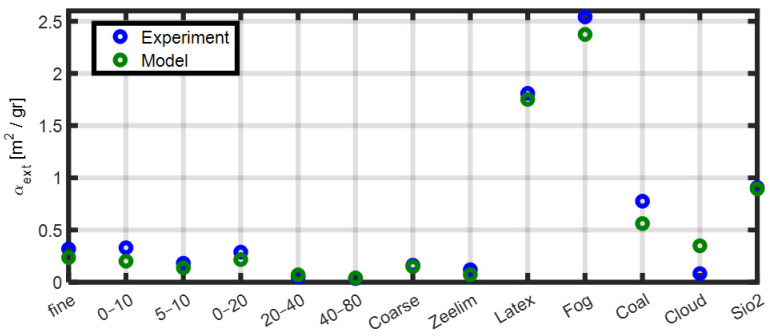
MEC at 650 nm for a range of aerosol types, obtained via Mie theory-based calculations (“Model”) and visibility sensor measurements (“Experiment”). The aerosol samples include different size fractions of Arizona Road Dust, natural desert dust (“Zeelim”), latex particles, fog droplets, carbonaceous particles, simulated water clouds, and silica powder.

MEC is a key parameter that characterizes the optical attenuation properties of an aerosol-laden medium. Figure 1 presents the calculated values of MEC for various aerosol types, including Arizona Road Dust (with different manufacturer-provided size distributions), Natural desert dust (collected in Zeelim, Israel), Fog machine aerosol, Water cloud droplets, Carbon particles, Latex microspheres, and Silica powder particles. This set of materials includes commonly used reference aerosols in atmospheric and optical research, representing well-characterized particle types differing in size, composition, and refractive index, allowing validation of the method against materials widely recognized in aerosol studies. Their size distribution functions, densities, and refractive indices are summarized in Table 1 and detailed in Appendix A.

1. MECs are shown as a comparison between two calculation methods: Computation from the measured particle size distributions using Equation (4). For each aerosol type, MEC was calculated by applying the appropriate density and the complex refractive index in the visible spectrum, as reported in the literature (“Model”). It should be noted that this method requires accurate measurement of the particle size distribution and spectral information on the refractive index of each material.
2. Estimation based on visibility range measurements, as detailed later in the Methods section (“Experiment”). This method is easy to implement since it requires no prior information and only a controlled calibration chamber.

The results presented in Figure 1 demonstrate good agreement between both approaches, supporting the validity of using visibility-derived extinction measurements for calibration purposes, on which the method presented in this work is based. Moreover, the data highlight that different aerosol types are characterized by distinctly different values of MEC, reflecting variations in particle properties such as size distribution and chemical composition.

The Meteorological Optical Range (MOR) provides an estimate of the density of an atmospheric medium using the Koschmieder equation [19]:

(5) $$Visibility=\frac{ln(20)}{\alpha_{ext}\cdot c}.$$

This equation establishes a direct relationship between the medium’s concentration *c* and the visibility range, mediated by MEC ($\alpha_{ext}$), and are both wavelength-dependent. Consequently, by measuring visibility and weighing the total aerosol mass, it is possible to calibrate an optical sensor to accurately determine the desired aerosol concentration. This provides a clear quantitative link between visibility-derived extinction and particulate mass concentration, through the mass extinction coefficient. Calibration of the optical sensor is performed by correlating the measured scattering intensity with aerosol mass concentration, using gravimetric reference data as the standard.

In order to assess the applicability of optical sampling for characterizing various aerosol materials, the scattering intensity was calculated for some materials that are widely employed in environmental research, listed in Table 1, based on Equation (3). This analysis aims to evaluate the sensitivity of LCPMSs or a visibility sensor to different aerosol types.

**Figure 2:**
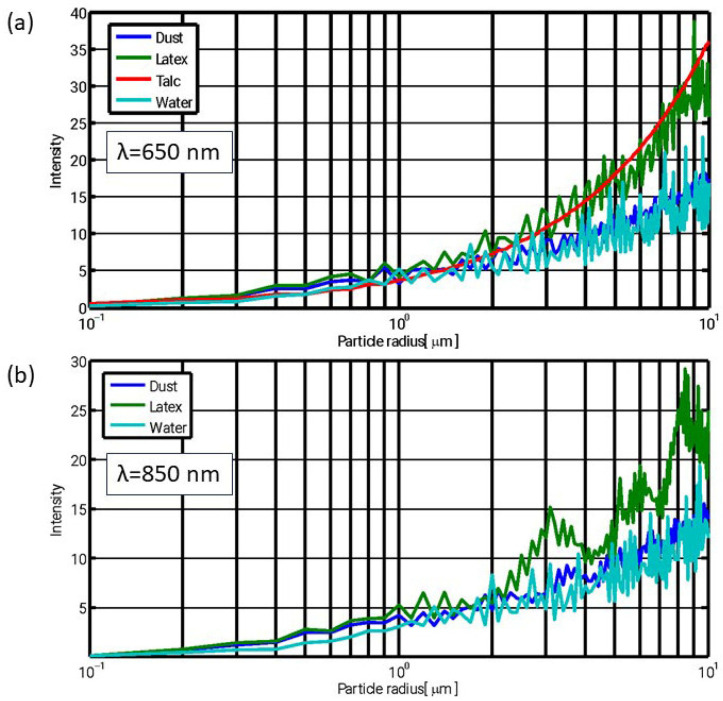
Calculated intensity of light scattered by a single particle at a 90° angle, for (**a**) λ = 650 nm and (**b**) 850 nm. Refractive indices and density values used in the calculations are listed in Table 1.

Figure 2 presents the calculated scattering intensity as a function of particle size, based on Equation (3), for two wavelengths representative of low-cost particulate matter sensors and visibility sensors.

**Figure 3:**
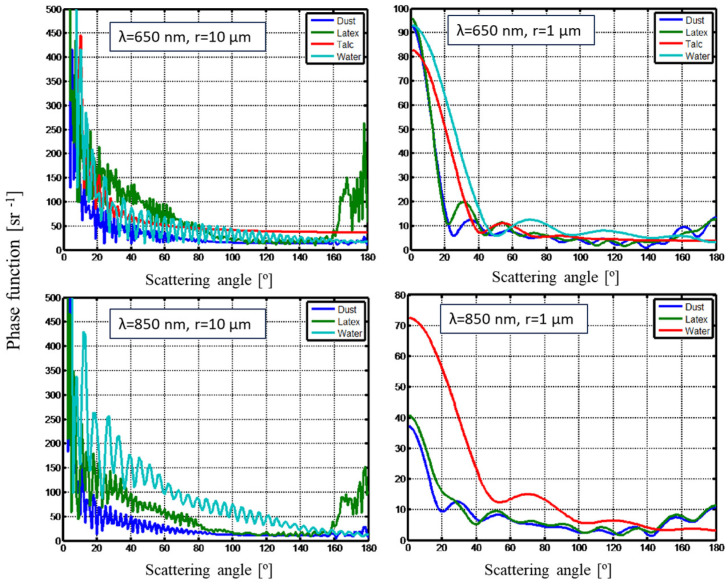
Scattering phase function for particles with radii of 1 µm and 10 µm, computed at wavelengths of 650 nm and 850 nm. The corresponding density and complex refractive index values used in the calculations are provided in Table 1.

As illustrated in Figure 2, the scattered intensity exhibits a systematic dependence on particle size, indicating that LCPMS, which detects individual scattering events, can infer both particle size (via signal amplitude) and number concentration over a broad range of materials. Notably, the similarity between the results for the two wavelengths suggests that comparable sensing performance can be achieved using either radiation source. Since the two measurement methods (visibility sensors and LCPMSs) operate at different wavelengths, their results are influenced differently by the particle-size-dependent light scattering. To illustrate this, the scattering phase function was computed for two particle sizes, multiple materials, and both wavelengths. The resulting comparison is shown in Figure 3.

Figure 3 demonstrates that at small (forward) scattering angles, the scattering intensity exhibits substantial material-dependent variation for both particle sizes. As the scattering angle increases, the overall intensity declines and inter-material differences diminish. These differences are more pronounced at 850 nm than at 650 nm. Since LCPMSs operate at visible wavelengths and non-forward angles, their measurements exhibit limited sensitivity to material composition. As a result, such sensors can be used as generalized detectors and calibrated using a universal, material-independent scaling factor.

Low-cost sensors are typically factory-calibrated using standard aerosol types, with their output correlated to that of reference-grade instruments [20]. This calibration is usually performed within predefined particle size categories, effectively anchoring the sensor response to the MEC of the calibration aerosol. Consequently, when deployed in environments containing aerosols with different optical properties, deviations in sensor output may occur. To mitigate artifacts stemming from variations in particle size distribution-particularly when comparing across aerosol types, calibration in this study is based solely on the PM_10_ channel, which integrates over the full particle size range.

Figure 3 also highlights that visibility sensors, operating at near-infrared wavelengths and detecting forward-scattered light, are more sensitive to aerosol composition. These instruments are typically calibrated under the assumption of water-based aerosols such as fog or rain. However, field studies report output deviations of up to 10–20% when exposed to natural aerosols with different optical characteristics [21]. Therefore, accurate application of visibility sensors requires initial calibration tailored to the aerosol type under investigation. Once calibrated, the visibility sensor can serve as a robust reference for calibrating LCPMS in similar environments.

### 2.2. Experimental Set-Up

**Figure 4:**
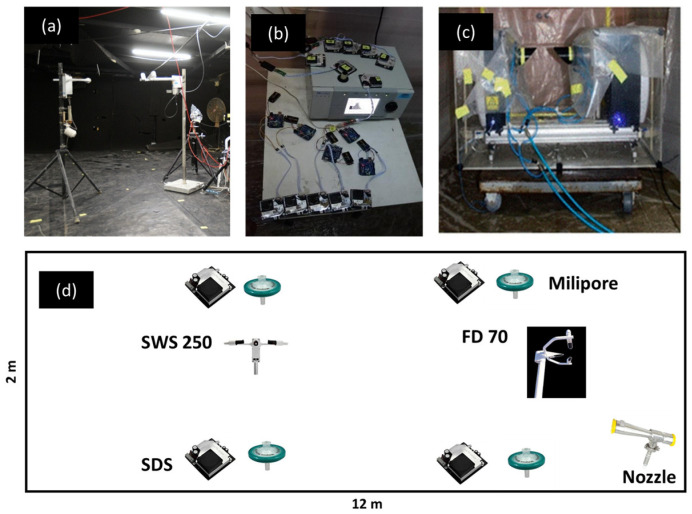
Experimental set-up. (**a**) FD70 and SWS250 visibility sensors; (**b**) SDS011 sensor network; (**c**) Spraytec laser diffraction analyzer; (**d**) Instrumentation layout inside the aerosol chamber.

All experiments were conducted in a sealed aerosol chamber; Figure 4 presents its layout. The low-cost optical sensors used were SDS011 units by Nova Fitness [22], which employ a 650 nm laser source. These commercial devices were integrated into a sensor network (Figure 4) and deployed at multiple positions within the chamber. The sensors measure particulate matter concentrations in size fractions PM_2.5_ and PM_10_, with an upper detection limit of 2 mg/m^3^.

Two visibility sensors were used, both operating on the forward-scatter principle, relating scattered light intensity to atmospheric extinction and visibility. The SWS250 [Biral, UK] operates in a forward-scatter geometry of about 45 degrees with a near-infrared light source at 880 nm and a measurement range from 10 m to 99.99 km, with a typical uncertainty of 10% at 10 km. The FD70 [Vaisala, Finland] uses a look-down forward-scatter geometry with a main receiver at 42 degrees and an auxiliary receiver at 90 degrees, illuminated by a near-infrared 850 nm source, covering a range from 3 m to 100 km with an uncertainty of ±10% up to 10 km. The FD70 offers enhanced temporal resolution and a low visibility detection threshold [23], making it particularly suitable for controlled chamber experiments. Visibility sensors were positioned to avoid spurious light reflections from chamber walls, as such reflections can affect the detected signal and alter the apparent visibility. In selected trials, aerosol particle size distributions were measured using a Spraytec laser diffraction system (Malvern Instruments, UK). Gravimetric measurements of total particulate matter concentration were obtained via Millipore air samplers. These systems use a suction pump to draw air through a filter, which captures airborne particles. The filters were weighed immediately after sampling using analytical balances, allowing accurate calculation of airborne mass concentration.

### 2.3. Calibration Method

The relationship between particulate matter concentration and meteorological optical range is governed by Equation (5). Establishing this relationship experimentally requires determination of the MEC for the aerosol under investigation.

While MEC can be computed directly using Equation (4), as shown in Figure 1, this method demands precise measurements of particle size distribution and accurate refractive index data across relevant wavelengths. As an alternative, MEC can be estimated empirically via gravimetric sampling combined with visibility sensor data. The strong agreement between the calculated and measured MEC values (Figure 1) supports the validity of this simplified calibration strategy.

Calibration was performed inside a sealed aerosol chamber under well-mixed conditions. As a representative case, we consider an experiment using a proline solution—an established plant stress marker [24]. Proline was selected as a stable and well-characterized compound that can be readily dispersed from aqueous solution, producing dry particles of consistent size after evaporation and thereby providing controlled conditions for calibration. The solution was atomized through using a spray nozzle, and subsequent droplet evaporation generated airborne particles with diameters ranging from 1 to 10 μm. These particles remained suspended for extended durations, enabling stable and repeatable measurements.

**Figure 5:**
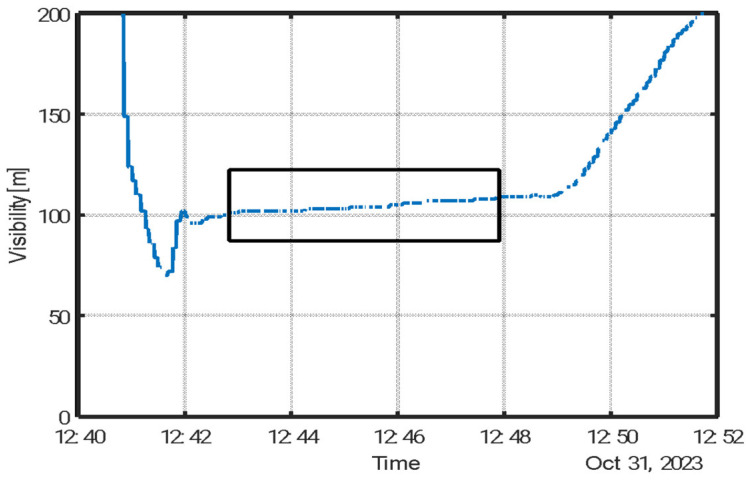
Visibility measurements (FD70) following dispersion of a 20% (*w*/*w*) proline solution. The sampling window is indicated by a black box. During this interval, the average particle mass concentration was 33 mg m^−3^, corresponding to an extinction coefficient of *α_ext_*(850 nm) = 0.88 m^2^ g^−1^.

Figure 5 depicts the experimental process, as recorded by a visibility sensor placed within the chamber. Initial atomization reduced visibility due to the high concentration of droplets and suspended particles. Following evaporation, only dry aerosol particles remained. Chamber mixing was then performed to ensure homogeneity, as indicated by the stabilization of the visibility signal. During this steady-state period (marked by a shaded region in Figure 5), aerosol samples were collected using Millipore filters.

MEC was subsequently derived from Equation (5), using the average visibility measured during the sampling window and the corresponding gravimetrically determined average mass concentration. Once the calibration constant is determined, PM concentration can be directly obtained from the measured optical extinction using Equation (5).

It is important to recognize that both visibility and MEC are wavelength-dependent, whereas mass concentration is not. For a given aerosol type, MEC determination is a one-time calibration. In repeated experiments where conditions remain constant, specifically, material type, particle size distribution, and humidity-visibility measurements can be directly converted to particulate mass concentration using Equation (5), with the extinction coefficient obtained from the initial calibration. However, any change in dispersion conditions or environmental parameters that affects the particle size distribution necessitates the recalculation of MEC.

Extension of the calibration to LCPMS involves simultaneous measurements with a visibility sensor. For clarity, the calibration workflow is summarized below, with each step yielding measurable results for the next stage.

1. Disperse the aerosol in a measurement chamber equipped with a visibility sensor. This step establishes the controlled environment for optical and gravimetric measurements.
2. After reaching the desired medium, ensure stable conditions (e.g., by mixing and verifying constant optical sensor readings). Stable readings indicate equilibrium of particle concentration and optical homogeneity, serving as the criterion for proceeding.
3. Under stable conditions, extract a sample for concentration determination by gravimetric analysis. This step yields the reference particulate mass concentration, averaged over several replicate samplings to confirm stability.
4. Calculate the mass extinction coefficient from the average visibility range during sampling and the measured concentration using Equation (5), with the extinction coefficient as the unknown. This calculation produces the MEC value [m^2^/g], which characterizes the optical properties of the aerosol type.
5. Assuming constant particle type and extinction coefficient, retrieve subsequent aerosol concentrations directly from visibility readings using Equation (5). This step provides estimated concentrations derived from optical measurements.
6. Define the calibration factor for the LCPMs as the ratio of the concentration derived from the visibility sensor to that measured by the LCPMs, enabling correction of LCPM readings according to particle type. This step yields the final calibration coefficient (*k*), used to adjust LCPM measurements for consistency with optical-derived concentrations, defined as $k=\frac{C_{\mathrm{vis}}}{C_{\mathrm{raw}}}$, where $C_{\mathrm{vis}}$ is the particulate concentration derived from visibility using Equation (5), and $C_{\mathrm{raw}}$ is the uncalibrated SDS011 output.

The implementation and validation of this calibration procedure are presented in the following section.

## 3. Results

**Figure 6:**
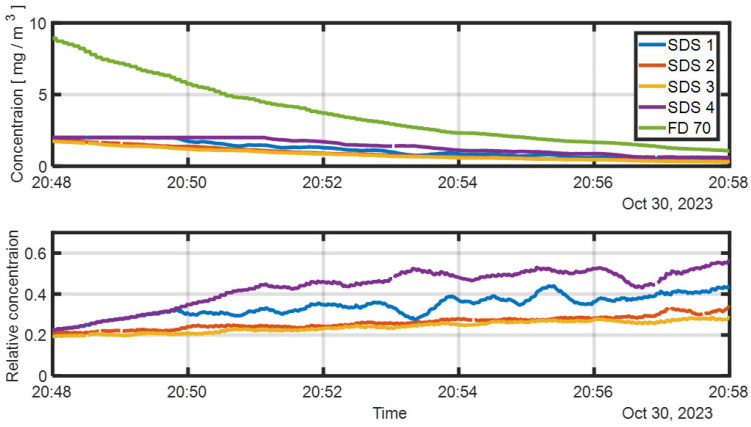
Time series of particulate concentration during an experiment with a 10% (*w*/*w*) proline solution. Top panel: Concentration estimated from the FD70 visibility sensor and raw PM_10_ readings from the SDS011 sensors, showing concentrations fall below threshold at 20:50; sensor readings stabilize after steady state is reached. Bottom panel: SDS011 raw measurements normalized relative to the visibility-derived concentration.

This section presents the calibration of SDS011 sensors using concurrent measurements from a visibility sensor. The upper panel of Figure 6 displays the raw signals from an array of four SDS011 sensors placed within the aerosol chamber, alongside the concentration inferred from the visibility sensor. Visibility-based concentration was calculated using Equation (5) with an MEC of 1.2 m^2^ g^−1^, determined from the average gravimetric (Millipore) measurement.

For calibration, the lower panel of Figure 6 shows the SDS011 readings normalized relative to the visibility-derived concentration. To carry out the calibration procedure as required, we focus on the raw concentration range appropriate for the SDS011 detector, i.e., concentrations below 2 mg/m^3^. In that range, the mean normalization factor was 0.4, with a standard deviation of 0.1, attributed to spatial concentration variability within the chamber. The low variance indicates a high degree of spatial homogeneity.

Multiplying the normalization factor by the MEC used in the visibility calculation yields an effective MEC for the SDS011 sensors of approximately 3 m^2^ g^−1^. This value reflects differences in the optical properties and operating wavelengths of the two sensor types.

**Figure 7:**
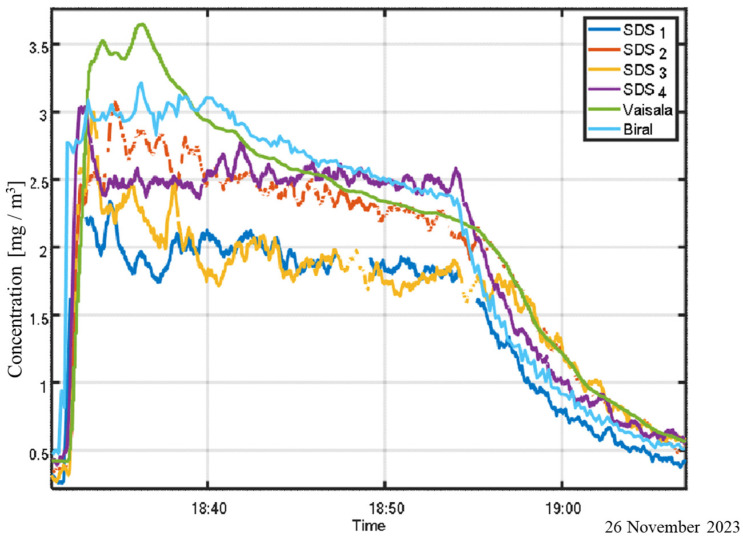
Calibrated sensor array used for spatial sampling of aerosol concentration within the chamber.

When experimental conditions are held constant, the calibration ratio remains stable and can be used to deploy a sensor network that accurately represents the chamber’s aerosol distribution with high spatial resolution, as illustrated in Figure 7. It can be seen that the calibration ratio leads to true concentrations that exceed the nominal threshold of the detectors.

**Figure 8:**
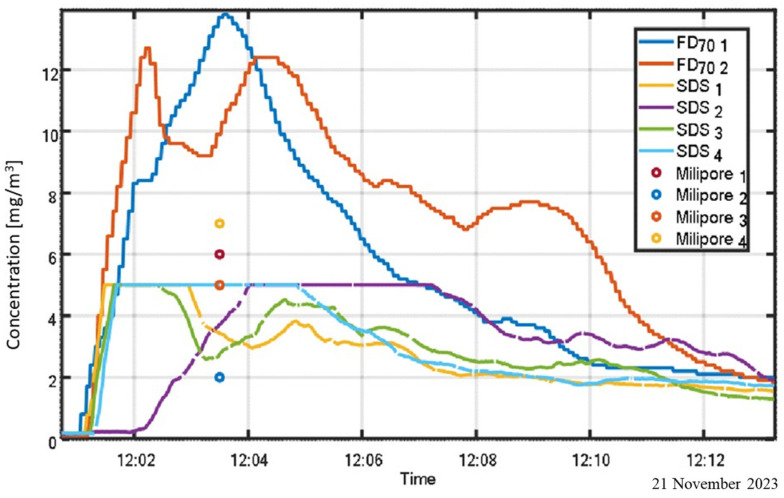
Comparison of particulate concentration measurements from calibrated visibility and SDS011 sensors, using Millipore gravimetric data as reference.

Figure 8 illustrates the benefits of calibrating optical sensors using visibility-based measurements, as opposed to relying solely on gravimetric sampling. The experiment involved three co-located instruments: a visibility sensor, an SDS011 sensor, and a Millipore gravimetric sampler, arranged side by side (see Figure 4).

By calibrating the visibility and SDS011 sensors against the gravimetric reference, continuous real-time monitoring of particulate concentration becomes feasible, assuming a stable particle size distribution during the measurement period. The figure also underscores the limitations of the SDS011 sensor, particularly its reduced upper detection limit relative to the visibility sensor.

## 4. Discussion

This study demonstrates the feasibility of calibrating low-cost optical sensors, such as the SDS011, for particulate matter concentration measurements using a visibility sensor as a reference.

Controlled experiments in sealed aerosol chambers enabled linking sensor outputs to actual mass concentrations by determining the MEC through parallel gravimetric sampling with Millipore filters. A key finding is that the experimentally derived MEC allows accurate and consistent conversion of sensor readings into mass concentrations, provided that aerosol properties and environmental conditions remain stable. SDS011 sensors are reliable below the manufacturer’s specified threshold limit and saturate above it. Notably, a substantial discrepancy was observed between factory-calibrated sensor responses and actual aerosol behavior, potentially leading to significant over- or underestimation during real-world operation, and also affecting the detection threshold. This discrepancy, evident in Figure 1, reflects the high sensitivity of MEC to aerosol composition- particularly particle size, density, and refractive index, emphasizing the need for aerosol-specific calibration.

In discussing the applicability of the proposed methodology, it is important to acknowledge its current limitations. The calibration experiments were conducted under controlled laboratory conditions, and not all measurements fell within the 0–2 mg/m^3^ operational range of the SDS011. Comprehensive validation under realistic atmospheric conditions remains necessary and is left for future work. In addition, particle generation was restricted to the 1–10 μm range, which is relevant for calibration but does not include fine and ultrafine fractions that are critical from a health perspective. The method has also not yet been validated across realistic atmospheric conditions, i.e., at lower concentration levels and higher visibility ranges. In addition, chamber experiments cannot account for environmental factors such as humidity, temperature, and variable aerosol composition, all of which can significantly influence both the MEC and the calibration stability of SDS011 sensors, as demonstrated in field-based studies [25,26]. Another important limitation is that experimental confirmation relied primarily on proline aerosols, whereas calibration for urban, traffic-derived, or biomass burning aerosols may be influenced by their differing composition, refractive index, and hygroscopic properties. Future investigations should therefore extend the validation to these aerosol types under realistic atmospheric conditions. Finally, when compared with nephelometer- or collocation-based approaches, the visibility-based calibration is inherently less accurate; however, it offers clear advantages in terms of cost, simplicity, and ease of deployment, thereby underscoring its potential as a practical tool for large-scale applications.

To frame the applicability and limitations of the method, we quantify the uncertainty associated with the proposed calibration, using relative standard uncertainties $u_{r}\left(x\right)=\frac{u(x)}{x}$. The overall uncertainty of the calibrated PM values was assessed by combining the partial uncertainties from all relevant instruments using standard error propagation methods. According to Equation (5), for MEC and the concentration retrieved from visibility, the combined relative uncertainties are as follows:

(6) $$\;\;\;\;\;\;\;\;\;\;\;\;\;\;\;\;\;\;\;\;\;\;\;\;\;\;\;\;\;\;\;\;\;\;u_{r}^{2}\left(MEC\right)=u_{r}^{2}\left(Visibility\right)+u_{r}^{2}\left(c_{grav}\right)+u_{r}^{2}\left(inhomogeniuity\right)+u_{r}^{2}\left(constants\right)\;u_{r}^{2}\left(c_{VIS}\right)=u_{r}^{2}\left(MEC\right)+u_{r}^{2}\left(Visibility\right)+u_{r}^{2}(constants).$$

Under the present laboratory conditions, the dominant contribution is the visibility measurement, with additional contributions from chamber spatial inhomogeneity and the gravimetric reference; the constant is treated as with negligible uncertainty. Specifically, we adopt $u_{r}\left(Visibility\right)\approx 0.04-0.10$ (in the low-visibility regime, instrument-dependent), $u_{r}\left(inhomogeniuity\right)\approx 0.05$ (from the spread of co-located sensors), $u_{r}\left(c_{grav}\right)\approx 0.04$ (from filter mass and flow calibration; timing is negligible). $u_{r}\left(constants\right)\approx 0$. Summing in quadrature yields $u_{r}\left(MEC\right)\approx 0.07-0.12$ and $u_{r}\left(c_{VIS}\right)\approx 0.09-0.16$, i.e., the relative uncertainty of the calibrated mass-extinction coefficient is estimated at ≈7–12%, and the concentration uncertainty of ≈9–16%.

The primary advantage of the proposed approach lies in its simplicity and practicality. Visibility sensors, already deployed at many meteorological stations, offer continuous and reliable optical measurements based on the same physical principles as low-cost sensors. This makes them ideal for establishing affordable calibration frameworks and dense monitoring networks, particularly in remote or resource-constrained settings.

Nonetheless, several limitations must be considered. First, the SDS011 exhibits a limited dynamic range, as confirmed by saturation effects in high-concentration chamber tests. Second, the visibility sensor itself requires initial calibration, typically based on gravimetric methods. Lastly, any changes in aerosol properties or environmental parameters may alter MEC, necessitating recalibration for accurate long-term deployment.

## 5. Conclusions

This study presents a theoretical framework and a laboratory demonstration of a practical and effective method for calibrating low-cost particulate matter sensors using visibility-based optical measurements. By leveraging the physical relationship between light extinction and aerosol concentration, as captured by the mass extinction coefficient (MEC), the proposed approach enables accurate translation of raw sensor data into mass concentration values. Experiments conducted in a controlled aerosol chamber demonstrate that the MEC can be reliably determined through concurrent gravimetric and visibility measurements. Once established for a given aerosol type under stable environmental conditions, the MEC allows both visibility sensors and SDS011 sensors to provide real-time, calibrated particulate matter readings. The consistency of the normalization factor across multiple SDS units highlights the potential for deploying dense sensor arrays with high spatial resolution.

The results also reveal that manufacturer calibrations of low-cost sensors may deviate significantly from actual aerosol behavior due to variations in particle size, composition, and optical properties. This underscores the need for application-specific calibration procedures.

This study is subject to several limitations, including validation restricted to laboratory conditions, the use of aerosols with a limited size range, and the use of proline aerosols as a representative case. Future work should therefore extend the calibration to diverse aerosol types and real atmospheric environments to establish broader applicability and robustness of the methodology. The proposed method offers a scalable alternative to traditional calibration approaches, requiring only a one-time gravimetric reference to establish the MEC. It is particularly well-suited for field deployments in resource-limited environments, where visibility sensors are already available and continuous monitoring is desired. The visibility-based approach, while less accurate than standard methods, offers clear advantages in cost and deployability.

## Figures and Tables

**Table 1:** Density and complex refractive index of Dust, Latex, Talc, and Water at Wavelengths of 650 nm and 850 nm (radiation source wavelengths of SDS011 and visibility sensor FD70, respectively).

Material	*m* @ 650 nm	*m* @ 850 nm	ρ [g cm^−3^]
Dust	1.53 + 0.008 i [13]	1.53 + 0.008 i [13]	2.6 [14]
Polystyrene	1.587 [15]	1.577 [14]	1.05 [16]
Talc	1.37 + 1.342 i [17]	N/A	2.75 [18]
Water	1.331 + 1.508 × 10^−8^ i [13]	1.3285 + 3.055 × 10^−7^ i [13]	1

**Figure A1:**
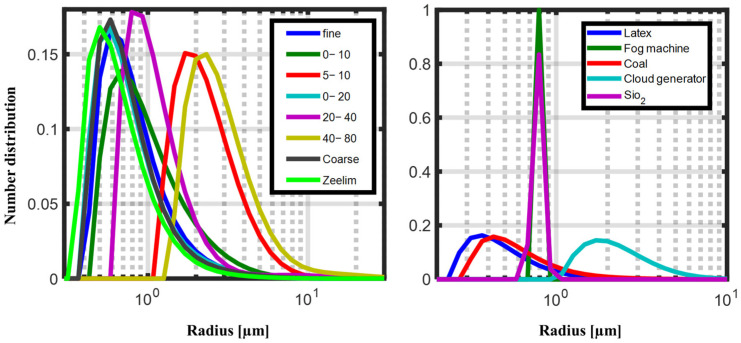
Normalized particle number distribution functions.

## Data Availability

The raw data supporting the conclusions of this article will be made available by the authors on request.

## Abbreviations

The following abbreviations are used in this manuscript:

LCPMS	Low-Cost Particulate Matter Sensors
PM	Particulate Matter
MEC	Mass Extinction Coefficient
MOR	Meteorological Optical Range

## Appendix A

This section presents the data used to calculate the MEC values shown in Figure 1. The normalized particle size distributions are displayed in Figure A1, while parameters not included in Table 1 are provided separately in Table A1.

**Table A1:** Density and complex refractive index of coal, fog, and SiO_2_ at wavelengths of 638 nm.

Material	*m*	ρ [g cm^−3^]
Coal	2.41 [27]	1.3 [28]
Fog machine liquid	1.41 [*]	1 [*]
SiO_2_	1.46 [29]	2 [30]

* The reported values represent the weighted average of the three constituents comprising a fog machine liquid, with weighting factors derived from NMR analyses.
